# Transcriptomic Characterization of Postmolar Gestational Choriocarcinoma

**DOI:** 10.3390/biomedicines9101474

**Published:** 2021-10-14

**Authors:** Constance Collet, Jonathan Lopez, Christophe Battail, Fabienne Allias, Mojgan Devouassoux-Shisheboran, Sophie Patrier, Nicolas Lemaitre, Touria Hajri, Jérôme Massardier, Benoit You, François Mallet, François Golfier, Nadia Alfaidy, Pierre-Adrien Bolze

**Affiliations:** 1 Institut National de la Santé et de la Recherche Médicale U1292, Biologie et Biotechnologie pour la Santé, 38043 Grenoble, France; constance.collet@cea.fr (C.C.); Christophe.BATTAIL@cea.fr (C.B.); nicolas.lemaitre@cea.fr (N.L.); nadia.alfaidy-benharouga@cea.fr (N.A.); 2 Commissariat à l’Energie Atomique et aux Energies Alternatives (CEA), Interdisciplinary Research Institute of Grenoble, CEDEX, 38054 Grenoble, France; 3 Service Obstétrique, Centre Hospitalo-Universitaire Grenoble Alpes, University Grenoble-Alpes, CS 10217, CEDEX 9, 38043 Grenoble, France; 4 Department of Biochemistry and Molecular Biology, Plateforme de Recherche de Transfert en Oncologie, University of Lyon 1, Hospices Civils de Lyon, University Hospital Lyon Sud, 165 Chemin du Grand Revoyet, 69495 Pierre Bénite, France; jonathan.lopez@chu-lyon.fr; 5 Centre de Recherche en Cancérologie de Lyon, INSERM U1052, CNRS UMR5286, Faculté de Médecine Lyon Est, 69008 Lyon, France; 6 Department of Pathology, University Hospital Lyon, Sud University of Lyon 1, Hospices Civils de Lyon, 165 Chemin du Grand Revoyet, 69495 Pierre Bénite, France; fabienne.allias@chu-lyon.fr (F.A.); mojgan.devouassoux@chu-lyon.fr (M.D.-S.); 7 French Center for Trophoblastic Diseases, University Hospital Lyon Sud, 165 Chemin du Grand Revoyet, 69495 Pierre Bénite, France; sophie.patrier@chu-rouen.fr (S.P.); touria.hajri@chu-lyon.fr (T.H.); jerome.massardier@chu-lyon.fr (J.M.); benoit.you@chu-lyon.fr (B.Y.); francois.golfier@chu-lyon.fr (F.G.); 8 Department of Pathology, University Hospital of Rouen, CEDEX, 76031 Rouen, France; 9 Department of Obstetrics and Gynecology, University Hospital Femme Mere Enfant, University of Lyon 1, 51, Boulevard Pinel, 69500 Bron, France; 10 Investigational Center for Treatments in Oncology and Hematology of Lyon (CITOHL), Medical Oncology Department, University of Lyon 1, Hospices Civils de Lyon, University Hospital Lyon Sud, 165 Chemin du Grand Revoyet, 69495 Pierre Bénite, France; 11 Joint Research Unit Hospices Civils de Lyon-bioMérieux, Hospices Civils de Lyon, Lyon Sud Hospital, 165 Chemin du Grand Revoyet, 69495 Pierre Bénite, France; francois.mallet@biomerieux.com; 12 Medical Diagnostic Discovery Department (MD3), bioMérieux S.A., 69280 Marcy l’Etoile, France; 13 Joint Research Unit Hospices Civils de Lyon-bioMérieux, EA 7426 Patho-Physiology of Injury-Induced Immunosuppression, PI3, Claude Bernard Lyon 1 University, Edouard Herriot Hospital, 69437 Lyon, France; 14 Department of Gynecological Surgery and Oncology, Hospices Civils de Lyon, University Hospital Lyon Sud, University of Lyon 1, Obstetrics, 165 Chemin du Grand Revoyet, 69495 Pierre Bénite, France

**Keywords:** gestational trophoblastic disease, gestational trophoblastic neoplasia, choriocarcinoma, hydatidiform mole, trophoblast, placenta, transforming growth factor beta

## Abstract

The human placenta shares properties with solid tumors, such as rapid growth, tissue invasion, cell migration, angiogenesis, and immune evasion. However, the mechanisms that drive the evolution from premalignant proliferative placental diseases—called hydatidiform moles—to their malignant counterparts, gestational choriocarcinoma, as well as the factors underlying the increased aggressiveness of choriocarcinoma arising after term delivery compared to those developing from hydatidiform moles, are unknown. Using a 730-gene panel covering 13 cancer-associated canonical pathways, we compared the transcriptomic profiles of complete moles to those of postmolar choriocarcinoma samples and those of postmolar to post-term delivery choriocarcinoma. We identified 33 genes differentially expressed between complete moles and postmolar choriocarcinoma, which revealed TGF-β pathway dysregulation. We found the strong expression of SALL4, an upstream regulator of TGF-β, in postmolar choriocarcinoma, compared to moles, in which its expression was almost null. Finally, there were no differentially expressed genes between postmolar and post-term delivery choriocarcinoma samples. To conclude, the TGF-β pathway appears to be a crucial step in the progression of placental malignancies. Further studies should investigate the value of TGF- β family members as biomarkers and new therapeutic targets.

## 1. Introduction

The human placenta shares some properties with solid tumors, such as rapid growth, tissue invasion, cell migration, angiogenesis, and immune evasion [1]. Whether these features of cancer emerged by selection or by the reactivation of embryonic pathways is currently unknown [1].

A recent study by Coorens et al. demonstrated that the normal human placenta is made up of clusters of tumor-like clonal expansions, yet it functions normally [2]. This study suggests that control processes might occur during placentation, but the underlying mechanisms are yet to be elucidated. Hence, studies assessing whether the genetic alterations seen in the neoplastic placenta, particularly in choriocarcinoma, are epigenetically driven could provide important insights into the mechanisms that accompany the development of this cancer.

As distinct from normal placental development, gestational trophoblastic diseases are a rare subset of placental conditions that include premalignant proliferations called partial or complete hydatidiform moles, and their invasive counterpart, named gestational trophoblastic neoplasia, of which choriocarcinoma is the most aggressive form. Complete sporadic moles mostly have diploid androgenetic monospermic genomes, with all the chromosomes originating from a haploid sperm and no maternal chromosomes [3]. Recently, Nguyen et al. showed that maternal bi-allelic deleterious mutations in the genes involved in meiotic double strand break formation, such as *MEI1*, might be involved in the pathogenesis of recurrent androgenetic complete moles. However, while it is well known that choriocarcinoma can derive from 2–3% of hydatidiform moles, the driving causes of this phenomenon remain unknown [4,5]. More rarely, choriocarcinoma may also develop after a normal pregnancy, with an incidence of 1 per 67,000 live births [6]. Choriocarcinoma following normal pregnancies are generally more severe and associated with an increased mortality compared to those arising from hydatidiform moles, but the determinants of its aggressiveness were poorly investigated [7].

It is well established that normal placental development strongly depends on the proliferation and invasion of trophoblast cells into the maternal decidua. These processes are controlled by autocrine and paracrine factors that ensure the fine cross talk between trophoblast cells and the cells that form the maternal decidua. The factors include those composing the large family of transforming growth factor (TGF) β. This family consists of a large group of growth and differentiation factors, including TGFβs, activins/inhibins, and bone morphogenetic proteins (BMPs). Importantly, the main members of the TGF-β family (TGF-β, bone morphogenetic protein (BMP), activin, and Nodal) play opposite roles in human placentation, either promoting or inhibiting trophoblast invasion. Although debatable, the majority of reports support the notion that TGF-β inhibits trophoblast invasion at the fetal–maternal interface, while BMP family members facilitate trophoblast invasion.

In relation to GTDs, the TGF-β signaling pathway plays an important role in the development and progression of gestational trophoblastic diseases, suggesting that members of this family may thus be employed as potential therapeutic targets and as diagnostic biomarkers [8].

Normal trophoblast cells are controlled by decidua-derived TGF-β, whereas choriocarcinoma cell lines are resistant to the antiproliferative as well as anti-invasive effects of TGF-β [9]. However, the nature of TGF-β signaling defects in the premalignant and malignant trophoblast remains unexplored.

To better understand the progression of placental tumorigenesis from premalignant molar pregnancies to their malignant counterparts, the choriocarcinoma, and the differences between a postmolar choriocarcinoma and post-term choriocarcinoma, we compared the transcriptomic profiles of complete hydatidiform moles and their subsequent choriocarcinoma, as well as the profiles of postmolar choriocarcinoma versus post-term choriocarcinoma. We used a “PanCancer Pathway panel” strategy that included 730 genes, among which the large TFG-β family was highly represented. The present study provides important translational data to develop diagnostic and therapeutic tools for placental diseases and cancer [10,11].

## 2. Material and Methods

### 2.1. Patients and Samples

Patients were registered in the French Reference Center for Trophoblastic Diseases, while the study (NCT03488901) was approved by the local ethical committee. Each histological diagnosis was confirmed by two referent pathologists from the Center. Samples were retrieved from the French Biobank for the study of Trophoblastic Diseases (CRB-HCL Hospices Civils de Lyon) [12].

To characterize each entity at a transcriptomic level, we compared the transcriptional profiles of 14 complete hydatidiform moles that subsequently transformed into choriocarcinoma after curettage to those of 17 postmolar choriocarcinomas. Hydatidiform and choriocarcinoma samples were paired for 12 patients. We then compared 17 postmolar to 20 post-term delivery choriocarcinoma samples. Patients were managed according to applicable clinical guidelines at the time of retrospective sample collection, i.e., with first-line monochemotherapy or polychemotherapy if the FIGO score was ≤6 or ≥7, respectively [13,14].

### 2.2. RNA Extraction

Macrodissection excluded peritumoral and necrotic tissue for choriocarcinoma samples and endometrium for hydatidiform mole samples. RNAs were extracted from formalin-fixed paraffin-embedded (FFPE) samples. Two to six 5 µm slides were used. The slides were first dewaxed with two baths of D-Limonene (2 min) and a bath of absolute ethanol (2 min), and RNA extraction was then performed using a High Pure FFPET RNA Isolation Kit (Roche, Switzerland, #06483852001).

### 2.3. Gene Expression

Gene expression analysis was conducted on the NanoString nCounter gene expression platform (NanoString Technologies, Inc., Seattle, WA, USA). We used a mixed code set consisting of a 730-gene panel (PanCancer Pathway) related to 13 cancer-associated canonical pathways (MAPK, STAT, PI3K, RAS, Cell Cycle, Apoptosis, Hedgehog, Wnt, DNA Damage Control, Transcriptional Regulation, Chromatin Modification, and the large TGF-β family) and a custom 30-gene panel (Supplementary Table S1) including those from the T cell-inflamed gene expression profile described by Ayers et al. [15] and trophoblast tolerance genes. Depending on concentrations, hybridization with Human PanCancer Progression probes (NanoString Technologies, USA, #XT-CSO-PROG1-12) was performed using 78–200 ng RNA, according to manufacturer’s instructions. After 17–20 h of incubation at 65 °C, the samples were processed on a NanoString nCounter FLEX platform. Raw counts from individual digital molecular barcodes were normalized on 6 positive internal controls and 40 housekeeping genes using nSolver 4.0 analysis software (NanoString Technologies, Inc., Seattle, WA, USA). The background was estimated from blank wells and six negative internal controls and was removed from raw counts.

### 2.4. Generation of Normalized Data

Each sample was analyzed in a separate multiplexed reaction, each including 8 negative probes and 6 serial concentrations of positive control probes. Negative control analysis was performed to determine the background for each sample.

Data were imported into nSolver analysis software (version 4.0, NanoString Technologies) for quality checking and normalization of the data. The first step of normalization using the internal positive controls permitted the correction of the potential variation associated with the technical workflow. We calculated the geometric mean of the positive probe counts for each sample. The scaling factor for a given sample was the ratio of the geometric mean of the sample to the average across all geometric means. For each sample, we divided all gene counts by the corresponding scaling factor. Finally, to normalize for differences in RNA input, we used the same method as was employed in positive control normalization, except that here, geometric means were calculated over 40 housekeeping genes (Supplementary Table S2). The results are expressed in fold change induction.

### 2.5. Principal Component Analysis

Principal component analysis (PCA) was performed using the sklearn.decomposition.PCA function in the Python package scikit learn (v0.22).

### 2.6. Differential Gene Expression Analysis

Normalized counts were analyzed according to the study’s objectives. The expression of every single gene within the categories was compared via t-test using the nSolver 4.0 software. The *p*-value and false discovery rate-adjusted *p*-value (Benjamini–Hochberg) were computed. Genes with an adjusted *p*-value < 0.05 and an absolute log2 fold change >1.0 were considered to be significantly differentially expressed.

### 2.7. Biological Pathway Enrichment Analysis

Biological pathway enrichments were performed on the significantly differentially expressed genes via the enrichGO function (FDR < 0.05) using the molecular function (MF) annotation tool in Gene Ontology GO.db_v3.10.0 (Bioconductor R3.6.3, https://www.bioconductor.org/, accessed on 27 August 2021). The enrichGO and the cnet (category net plot used for visualization) functions were executed by ClusterProfiler v3.14.3 (Bioconductor R3.6.3, https://www.bioconductor.org/, accessed on 27 August 2021).

### 2.8. Immunohistochemistry

Tissue samples were processed as described previously [16]. In total, 14 complete mole and 15 postmolar choriocarcinoma samples were included for immunohistochemical analysis. Monoclonal Sall4 antibody (Sigma–Aldrich, 38070 Saint Quentin Fallavier, France) was used at 0.2 µg/mL. To quantify the intensity of the immunostainings, the images were morphometrically analyzed using Image J software.

## 3. Results

### 3.1. Comparison of Complete Moles versus Postmolar Choriocarcinoma

#### 3.1.1. Clinical Characteristics

The clinical characteristics of patients with a diagnosis of complete mole and/or postmolar choriocarcinoma are presented in Table 1. As expected, most of the postmolar choriocarcinoma patients displayed low-risk disease (i.e., FIGO score ≤6) limited to the pelvis (i.e., FIGO stage I or II), and were treated via monochemotherapy or surgery.

#### 3.1.2. Differential Gene Expression between Complete Mole and Postmolar Choriocarcinoma

**Figure 1:**
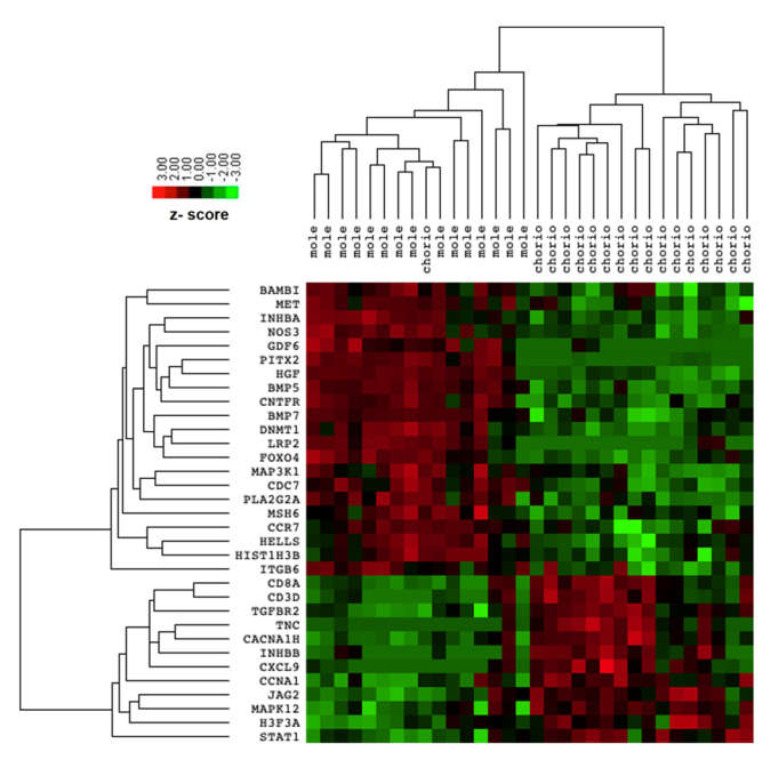
Heatmap of differentially expressed genes between complete mole and postmolar choriocarcinoma.

The comparison between transcriptomic profiles of complete mole and postmolar choriocarcinoma samples identified 33 differentially expressed genes (DEG) with an adjusted FDR < 0.05, as presented in Table 2. Among these 33 DEG, 12 were upregulated and 21 downregulated in the postmolar choriocarcinoma stage. The samples were clustered according to disease stage. Postmolar choriocarcinoma was substantially different from the complete mole, which was clustered as one dendrogram (indicated by the DEG), except for one choriocarcinoma sample that was clustered with a complete mole (Figure 1).

#### 3.1.3. Pathway Analysis

**Figure 2:**
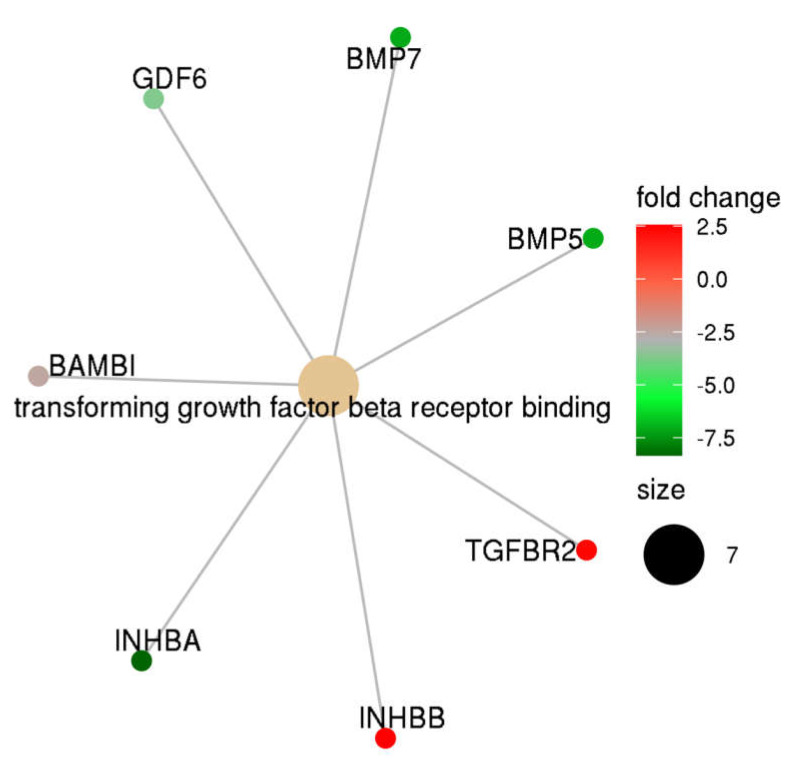
TGF-β family members’ expression profiles in postmolar choriocarcinoma when compared to that of complete hydatidiform moles.

Gene set enrichment analysis was performed using the DEG list presented in Table 2. Using a stringent threshold (FDR < 0.05), we identified that the TGF-β receptor binding pathway was significantly different between complete mole and postmolar choriocarcinoma entities (Figure 2). Indeed, TGF-β network analysis showed that, in postmolar choriocarcinoma, BMP5, BMP7, INHB-A, and GDF6 were largely underexpressed, while TGF-β receptor 2 and INH-B were overexpressed when compared with that of the complete mole.

#### 3.1.4. TGF-β Upstream Analysis

**Figure 3:**
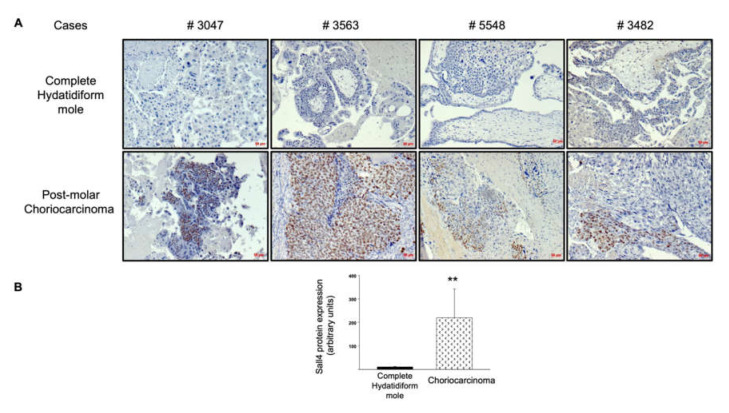
SALL4 protein expression in complete mole and postmolar choriocarcinoma. (**A**) Comparison of SALL4 immunostaining on paired samples. (**B**) Quantitative assessment of SALL4 expression via immunohistochemistry. ** *p* < 0.01 ± SEM. Scale bar = 50 µm.

Next, we explored the upstream regulation of the TGF-β receptor pathway. Given the role of SALL4 in the activation of the TGF-β/SMAD signaling pathway to promote epithelial–mesenchymal transition and metastasis in other cancers, and the upregulation described in gestational choriocarcinoma, we assessed SALL4 protein expression in complete mole and postmolar choriocarcinoma samples. SALL4 was expressed in the cytotrophoblast of most postmolar choriocarcinoma samples, with heterogeneity among samples, while almost none of the complete mole samples showed SALL4 immunostaining (Figure 3A). The immunostaining digital quantitative assessment visually confirmed SALL4 overexpression in postmolar choriocarcinoma compared to that of complete mole samples (Figure 3B).

### 3.2. Comparison of Postmolar Choriocarcinoma versus Post-Term Delivery Choriocarcinoma

The clinical characteristics of patients with postmolar and post-term delivery are presented in Table 3. Patients with post-term delivery choriocarcinoma displayed more advanced disease (FIGO stage III and IV), defined by lung (stage III) and liver or brain (stage IV) metastasis. Of the 20 samples taken from post-term delivery choriocarcinoma, 10 were collected from patients with fatal evolution.

#### Differential Gene Expression between Postmolar Choriocarcinoma and Post-Term Delivery Choriocarcinoma

The comparison between the transcriptomic profiles of postmolar choriocarcinoma and post-term delivery choriocarcinoma samples did not identify differentially expressed genes (DEG) with an adjusted FDR < 0.05. Only three DEG with an FDR < 0.25 were identified (Table 4). MSH2 was slightly overexpressed, while LTBP1 and RAC1 were underexpressed, in post-term delivery choriocarcinoma when compared to that of postmolar choriocarcinoma. Due to the very limited number of DEG and their elevated FDR, we did not conduct pathway analysis for this comparison.

## 4. Discussion

In the present study, the PanCancer transcriptomic profiles used did not show any significant differences between postmolar and post-term choriocarcinoma; however, significant differences were observed, especially in the TGF-β large family, between complete molar pregnancies and subsequent postmolar choriocarcinoma.

These results strongly suggest that term choriocarcinoma, despite being associated with a worse prognosis, should be considered from a transcriptomic point of view, similarly to postmolar choriocarcinoma, at least regarding the present analysis. Nevertheless, the enrichment analysis used in this study employed predesigned genes, which suggests that if a larger panel of genes was considered, the analysis would have revealed significantly deregulated genes and/or pathways. Because the present study compared postmolar choriocarcinoma to term-choriocarcinoma at the transcriptomic level, this does not exclude potential differential expression and or function of tumor-associated proteins. Hence, a similar study that compares the proteome of both entities may provide useful insights into the underlying mechanism of development of these two tumors. Hence, the bad prognosis associated with term choriocarcinoma may be explained by other factors, such as the increased delay in the diagnosis of a post-term choriocarcinoma compared to postmolar choriocarcinoma. Indeed, postmolar surveillance (i.e., weekly serum hCG) is much more intense than the surveillance following term delivery, where patients usually do not undergo routine hCG monitoring [17,18,19]. Also, according to the FIGO score, post-term choriocarcinoma are diagnosed at stages much more advanced than postmolar choriocarcinoma. This may largely explain the observed differences in their prognostic that is substantiated by difference in the death number, which is 10 times higher in patients with post-term choriocarcinoma compared to postmolar choriocarcinoma.

Because of the scarcity of choriocarcinoma, and the difficulty of collecting samples at two different time points from the same patient so as to compare CHM and postmolar CC, the present cohort offer highly valuable information. Thanks to this collection and despite the high variability, we were able to identify a significant number of differentially expressed genes. The transcriptomic analysis of complete molar pregnancies and their subsequent choriocarcinoma revealed significant differential changes in the expressions of numerous key placental genes. A total of 33 genes were differently expressed; 21 were upregulated in the postmolar choriocarcinoma condition, and 12 were downregulated.

Among the downregulated genes, we identified several members of the BMP family, such as BMP5, BMP7, and GDF6, and some of the activin/inhibin family, such as INHBA. Most of the upregulated genes belonged to the TGF-β family, including its receptor, TGF-β-R2.

The reduced expression of the members of the BMP family in the choriocarcinoma samples strongly suggests that these genes play suppressive roles in this type of cancer. The inhibitory role of BMPs in tumorigenesis and dissemination was widely reported in previous studies. For instance, BMP7 was reported to function as a potent tumor suppressor in gastric carcinoma, renal cell carcinoma, lung and colorectal cancer, and osteosarcoma. In these cancers, BMP7 suppresses tumor growth by reducing the gene expression of tumorigenic factors and by inducing the differentiation of cancer stem cells [20]. Additionally, several studies demonstrated that BMP5 functions as a tumor suppressor in myeloma, adrenocortical carcinoma, and breast cancer [21]. In line with these findings, the BMP5 gene is decreased in colorectal carcinoma (CRC) and plays an inhibitory role in controlling the associated metastases [22]. Importantly, the loss of BMP signals was cited as one of the two main genetic alterations leading to CRC, as disrupted BMP signaling allows tumor growth and expansion [22].

In relation to the TGF-β family, we observed an increase in the levels of the expression of some of its members in postmolar choriocarcinoma samples compared to those observed at the complete mole stage. This result is in line with a previous study demonstrating that TGF-β signaling is required to accelerate tumor cell invasion, through a process involving epithelial to mesenchymal transition [23].

Importantly, we demonstrated that the choriocarcinoma-associated transcription factor Sall4 was increased in situ in the postmolar choriocarcinoma cohort compared to that of the complete mole counterpart. This finding is in line with previous studies demonstrating that Sall4 plays a key role in tumorigenesis and tumor cell invasiveness through its correlation with TGF-β signaling genes [24,25].

Furthermore, Sall4 is specifically expressed by cancer cells in choriocarcinoma [26]. The observation of a strong increase in SALL4-positive cells as the complete hydatidiform mole progresses into cancer further supports our genetic analysis, and the assumption that this signaling cascade is involved in the development of choriocarcinoma from CHM.

As such, one can also speculate that the increase in TGF-β signaling may occur prior to the increase in TGF-β sensitivity during the evolution from complete mole to choriocarcinoma, which may make trophoblast cells hyper-proliferative and thus more prone to further invasion and mutational events.

To date, the complex role of TGF-β signaling in relation to tumorigenesis was well documented, and sequential stages were proposed. In the early stages of the disease, this signaling mainly has tumor-suppressive effects via cell cycle inhibition and apoptosis induction. Throughout cancer progression, these inhibitory effects are lost, and its role switches to support tumor growth and metastatic processes [27]. Therefore, the global increase in genes belonging to the TGF-β family when choriocarcinoma develops from the choriocarcinoma stage suggests that TGF-β-associated signaling might be a key driver of cancer development.

Taken together, these results strongly support the assumption that the large family of TGF-β (TGF-β, BMP and activin/inhibin) plays dual roles in gestational trophoblastic diseases, and that the dual actions may depend on the stage of the pathology. This large family may contribute to the transition from a pre-malignant to a malignant form of placental tumor.

We propose that TGF-β signaling should be considered as a key pathway in the pathogenesis and progression of gestational trophoblastic disease, and may thus be exploited as a potential therapeutic target and diagnostic biomarker. However, to date, none of the attempts made to predict postmolar malignant transformation via transcriptomic methods succeeded [5,28]. Whole-transcriptome and epigenome approaches might complement the present conclusions regarding the involvement of TGF-β in the malignant transformation of complete moles.

## Figures and Tables

**Table 1:** Clinical characteristics of patients with complete mole and/or postmolar choriocarcinoma.

	Complete Mole	Postmolar Choriocarcinoma
	*n* = 14	*n* = 17
Age (mean, range), y	37 (25–55)	35 (26–55)
Serum hCG before D&C		
FIGO score (median, range)	-	3 (0–8)
hCG at treatment initiation (median, range); IU/L	-	19,098 (739–201,938)
Larger tumor size >5 cm (*n*)	-	3
FIGO stage (*n*)	-	
I	-	14
II	-	1
III	-	2
First-line treatment (*n*)	-	
Monochemotherapy (methotrexate)	-	9
Polychemotherapy (EMA-CO)	-	5
Surgery (hysterectomy)	-	3

hCG, human chorionic gonadotropin; IU/L, international units/liter; D&C, dilatation and curettage; FIGO, Fédération Internationale des Gynécologues et Obstétriciens; EMA-CO, etoposide, methotrexate and actinomycin-D alternated weekly with cyclophosphamide and vincristine.

**Table 2:** Differentially expressed genes between complete hydatidiform mole and postmolar choriocarcinoma samples (FDR < 0.05).

Gene Name	Relative Expression Fold Change	FDR Adjusted *p*-Value
BMP5	−7.08	0
BMP7	−7.12	0
CDC7	−1.97	0
CNTFR	−6.08	0
DNMT1	−2.53	0
GDF6	−3.69	0
HGF	−26.08	0
INHBA	−8.34	0
LRP2	−10.54	0
NOS3	−4.75	0
PITX2	−9.83	0
BAMBI	−2.46	0.01
CACNA1H	5.53	0.01
CCNA1	2.04	0.01
CD8A	4.19	0.01
FOXO4	−2.54	0.01
HELLS	−1.92	0.01
MET	−3.77	0.01
TGFBR2	2.39	0.01
TNC	4.04	0.01
H3F3A	1.36	0.02
JAG2	2.35	0.02
MAPK12	2.43	0.02
PLA2G2A	−8.05	0.02
HIST1H3B	−2.54	0.03
INHBB	2.55	0.03
MAP3K1	−1.29	0.03
MSH6	−1.37	0.03
STAT1	1.98	0.03
CCR7	−3.38	0.04
CD3D	3.12	0.04
CXCL9	5.86	0.04
ITGB6	−2.3	0.04

**Table 3:** Clinical characteristics of patients with postmolar and post-term-delivery choriocarcinoma.

	Postmolar Choriocarcinoma	Post-Term Delivery Choriocarcinoma
	*n* = 17	*n* = 20
Age (mean, range), y	35 (26–55)	31 (23–45)
FIGO score (median, range)	3 (0–8)	9.5 (3–17)
Interval since antecedent pregnancy termination >12 months (*n*)	6	6
hCG at treatment initiation (median, range); IU/L	19,098 (739–201,938)	39,069 (735–479,771)
Larger tumor size >5cm (*n*)	3	10
Liver or brain metastasis (*n*)	0	
FIGO stage (*n*)		
I	14	9
II	1	0
III	2	8
IV	0	3
First-line treatment (*n*)		
Monochemotherapy (methotrexate)	9	7
Polychemotherapy (EMA-CO)	5	13
Surgery (hysterectomy)	3	0
Death (*n*)	0	10

FIGO, Fédération Internationale des Gynécologues et Obstétriciens; hCG, human chorionic gonadotropin; IU/L, international units/liter; D&C, dilatation and curettage; EMA-CO, etoposide, methotrexate and actinomycin-D alternated weekly with cyclophosphamide and vincristine.

**Table 4:** Differentially expressed genes between postmolar choriocarcinoma and post-term delivery choriocarcinoma samples (FDR < 0.25).

Gene Name	Relative Expression Fold Change	FDR Adjusted *p*-Value
MSH2	1.58	0.08
LTBP1	−1.97	0.09
RAC1	−1.29	0.09

## Supplementary Materials

The following are available online at https://www.mdpi.com/article/10.3390/biomedicines9101474/s1—Supplementary Table S1: Custom gene panel; Supplementary Table S2: Housekeeping genes.
