# Supplementary material for: Transcriptomic Characterization of Postmolar Gestational Choriocarcinoma
Source: Biomedicines. 2021 Oct 14;9(10):1474. doi: 10.3390/biomedicines9101474 (PMC8533618; doi:10.3390/biomedicines9101474)
Supplement: Supplementary file 1 [file biomedicines-09-01474-s001.zip › Supplementary Table S1.pdf]

Supplementary table S1

Gene name	Accession	Position	Target sequence
CCL5	NM_002985.2	281-380	AGTGTGTGCCAACCCAGAGAAGAAATGGGTTCGGGAGTACATCAACTCTTTGGAGATGAGCTAGGATGGAGAGTCCTTGAACCTGAACTTACACAAATTT
CD2	NM_001767.3	688-787	GCAGCCTCTTGATGGTCTTTGTGGCACTGCTCGTTTTCTATATCACCAAAAGGAAAAAACAGAGGAGTCGGAGAAATGATGAGGAGCTGGAGACAAGAGC
CD27	NM_001242.4	1102-1201	GTCAGGGCCCTTTCCTGTGTACACGTGACAGAGTGCCTTTTCGAGACTGGCAGGGACGAGGACAAATATGGATGAGGTGGAGAGTGGGAAGCAGGAGCCC
CD274	NM_014143.3	50-149	AGCTTCCCGAGGCTCCGCACCAGCCGCGCTTCTGTCCGCCTGCAGGGCATTCCAGAAAGATGAGGATATTTGCTGTCTTTATATTCATGACCTACTGGCA
CD276	NM_001024736.1	2121-2220	ACATTTCTTAGGGACACAGTACACTGACCACATCACCACCCTCTTCTTCCAGTGCTGCGTGGACCATCTGGCTGCCTTTTTTCTCCAAAAGATGCAATAT
CD3D	NM_000732.4	111-210	TATCTACTGGATGAGTTCCGCTGGGAGATGGAACATAGCACGTTTCTCTCTGGCCTGGTACTGGCTACCCTTCTCTCGCAAGTGAGCCCCTTCAAGATAC
CD8A	NM_001768.5	1321-1420	GCTCAGGGCTCTTTCCTCCACACCATTCAGGTCTTTCTTTCCGAGGCCCCTGTCTCAGGGTGAGGTGCTTGAGTCTCCAACGGCAAGGGAACAAGTACTT
CIITA	NM_000246.3	3048-3147	CTTTCCCCAAACTGGTGCGGATCCTCACGGCCTTTTCCTCCCTGCAGCATCTGGACCTGGATGCGCTGAGTGAGAACAAGATCGGGGACGAGGGTGTCTC
CMKLR1	NM_004072.1	771-870	CAACGTCTTCCTCCCAATCCATATCACCTATGCCGCCATGGACTACCACTGGGTTTTCGGGACAGCCATGTGCAAGATCAGCAACTTCCTTCTCATCCAC
CTLA-4	NM_005214.3	406-505	AGTCTGTGCGGCAACCTACATGATGGGGAATGAGTTGACCTTCCTAGATGATTCCATCTGCACGGGCACCTCCAGTGGAAATCAAGTGAACCTCACTATC
CXCL10	NM_001565.2	462-561	GCCATAATTGTTCTTAGTTTGCAGTTACACTAAAAGGTGACCAATGATGGTCACCAAATCAGCTGCTACTACTCCTGTAGGAAGGTTAATGTTCATCATC
CXCL13	NM_006419.2	211-310	AGACGCTTCATTGATCGAATTCAAATCTTGCCCCGTGGGAATGGTTGTCCAAGAAAAGAAATCATAGTCTGGAAGAAGAACAAGTCAATTGTGTGTGTGG
CXCL9	NM_002416.1	1976-2075	CACCATCTCCCATGAAGAAAGGGAACGGTGAAGTACTAAGCGCTAGAGGAAGCAGCCAAGTCGGTTAGTGGAAGCATGATTGGTGCCCAGTTAGCCTCTG
CXCR6	NM_006564.1	96-195	TTACCATGAAGACTATGGGTTCAGCAGTTTCAATGACAGCAGCCAGGAGGAGCATCAAGACTTCCTGCAGTTCAGCAAGGTCTTTCTGCCCTGCATGTAC
ERVW-1	NM_014590.3	1991-2090	AAGAGTACCCATTCTTCCTTTTGTTATAGGAGCAGGAGTGCTAGGTGCACTAGGTACTGGCATTGGCGGTATCACAACCTCTACTCAGTTCTACTACAAA
HAVCR2	NM_032782.3	956-1055	TATATGAAGTGGAGGAGCCCAATGAGTATTATTGCTATGTCAGCAGCAGGCAGCAACCCTCACAACCTTTGGGTTGTCGCTTTGCAATGCCATAGATCCA
HLA-DQA1	NM_002122.3	262-361	GGTGGCCTGAGTTCAGCAAATTTGGAGGTTTTGACCCGCAGGGTGCACTGAGAAACATGGCTGTGGCAAAACACAACTTGAACATCATGATTAAACGCTA
HLA-DRA	NM_019111.3	336-435	GGCCAACATAGCTGTGGACAAAGCCAACCTGGAAATCATGACAAAGCGCTCCAACTATACTCCGATCACCAATGTACCTCCAGAGGTAACTGTGCTCACG
HLA-DRB1	NM_002124.3	748-847	AGCACGGTCTGAATCTGCACAGAGCAAGATGCTGAGTGGAGTCGGGGGCTTTGTGCTGGGCCTGCTCTTCCTTGGGGCCGGGCTGTTCATCTACTTCAGG
HLA-E	NM_005516.4	1205-1304	TGTCTTAGGGGACTCTGGCTTCTCTTTTTGCAAGGGCCTCTGAATCTGTCTGTGTCCCTGTTAGCACAATGTGAGGAGGTAGAGAAACAGTCCACCTCTG
HLA-G	NM_002127.4	1181-1280	AAGAGCTCAGATTGAAAAGGAGGGAGCTACTCTCAGGCTGCAATGTGAAACAGCTGCCCTGTGTGGGACTGAGTGGCAAGTCCCTTTGTGACTTCAAGAA
IDO1	NM_002164.3	51-150	CTATTATAAGATGCTCTGAAAACTCTTCAGACACTGAGGGGCACCAGAGGAGCAGACTACAAGAATGGCACACGCTATGGAAAACTCCTGGACAATCAGT
LAG3	NM_002286.5	1736-1835	CTTTTGGTGACTGGAGCCTTTGGCTTTCACCTTTGGAGAAGACAGTGGCGACCAAGACGATTTTCTGCCTTAGAGCAAGGGATTCACCCTCCGCAGGCTC
NCAM1	NM_000615.5	1621-1720	GGTATTTGCCTATCCCAGTGCCACGATCTCATGGTTTCGGGATGGCCAGCTGCTGCCAAGCTCCAATTACAGCAATATCAAGATCTACAACACCCCCTCT
NKG7	NM_005601.3	633-732	CTGTGGCGGTCCCCGTCCTGGCTATGAAACCTTGTGAGCAGAAGGCAAGAGCGGCAAGATGAGTTTTGAGCGTTGTATTCCAAAGGCCTCATCTGGAGCC
PDCD1	NM_005018.2	311-410	CTTCCCCGAGGACCGCAGCCAGCCCGGCCAGGACTGCCGCTTCCGTGTCACACAACTGCCCAACGGGCGTGACTTCCACATGAGCGTGGTCAGGGCCCGG
PDCD1LG2	NM_025239.3	236-335	TGTGGAGCTGTGGCAAGTCCTCATATCAAATACAGAACATGATCTTCCTCCTGCTAATGTTGAGCCTGGAATTGCAGCTTCACCAGATAGCAGCTTTATT
PSMB10	NM_002801.2	222-321	ACCATCGCGGGCCTGGTGTTCCAAGACGGGGTCATTCTGGGCGCCGATACGCGAGCCACTAACGATTCGGTCGTGGCGGACAAGAGCTGCGAGAAGATCC
TIGIT	NM_173799.2	1969-2068	TGGATCTTAGAAGACTTTTATCCTTCCACCATCTCTCTCAGAGGAATGAGCGGGGAGGTTGGATTTACTGGTGACTGATTTTCTTTCATGGGCCAAGGAA
VISTA (VSIR)	NM_022153.1	1956-2055	CTGGGACACTTCTGAGTATGAAGCGGGATGCTATTAAAAACTACATGGGGAAACAGGTGCAAACCCTGGAGATGGATTGTAAGAGCCAGTTTAAATCTGC

Supplementary table S1. Custom gene panel.
